# Supplementary material for: Early intervention in psychosis programs in Africa, Asia and Latin America; challenges and recommendations
Source: Glob Ment Health (Camb). 2025 Jan 6;12:e3. doi: 10.1017/gmh.2024.78 (PMC11704378; doi:10.1017/gmh.2024.78)
Supplement: van der Ven et al. supplementary material [file S2054425124000785sup001.docx]

**Supplementary material**

**Ovid Medline Search Results – 06 Feb 2024**

| **Search** | **Query** | **Results** |
| --- | --- | --- |
| #5 | **3 and 4** | 332 |
| #4 | **"Developing Countries"/ or ("developing countr*" or "non-western" or "nonwestern" or "emerging econom*" or "developing nation*" or "developing population*" or "developing econom*" or "undeveloped countr*" or "undeveloped nation*" or "undeveloped economy" or "undeveloped economies" or "least developed countr*" or "least developed nation*" or "least developed economy" or "least developed economies" or "less-developed countr*" or "less-developed nation*" or "less-developed population" or "less-developed populations" or "less-developed econom*" or "lesser developed countr*" or "lesser developed nation*" or "lesser developed population" or "lesser developed populations" or "lesser developed economy" or "lesser developed economies" or "under-developed countr*" or "under-developed nation*" or "underdeveloped countr*" or "underdeveloped nation*" or "underdeveloped population*" or "underdeveloped econom*" or "low income countr*" or "middle income countr*" or "low income nation*" or "middle income nation*" or "low income population*" or "middle income population*" or "low income econom*" or "middle income econom*" or "lower income countr*" or "lower income nation*" or "lower income population*" or "lower income economy" or "lower income economies" or "resource limited" or "low resource countr*" or "lower resource countr*" or "low resource nation*" or "low resource population*" or "low resource economy" or "low resource economies" or "underserved countr*" or "underserved nation*" or "underserved population*" or "underserved economy" or "underserved economies" or "under-served country" or "under-served countries" or "under-served nation" or "under-served nations" or "under-served population" or "under-served populations" or "underserved economy" or "underserved economies" or "derived countr*" or "deprived nation" or "deprived nations" or "derived population*" or "deprived economy" or "deprived economies" or "poor countr*" or "poor nation*" or "poor population*" or "poor econom*" or "poorer countr*" or "poorer nation*" or "poorer population*" or "poorer econom*" or "lmic" or "lmics" or "lami" or "transitional countr*" or "transitional nation" or "transitional nations" or "transitional econom*" or "transition countr*" or "transition nation*" or "transition econom*" or "low resource setting*" or "lower resource setting*" or "middle resource setting*" or "Third World*" or Afghan* or Alban* or Algeria* or Angol* or Argentin* or Armenia* or Azerbaijan* or Bangladesh* or Belarus* or Belize* or Benin or Bhutan* or Bolivia* or Bosnia* or Botswan* or Brazil* or "Burkina Faso" or Burundi* or "Cabo Verd*" or Cambodia* or Cameroon* or "Central African Republic" or Chad or China or Colombia* or Comoros or Congo* or "Costa Rica*" or "Côte d’Ivoire" or Cuba* or "Republic of Korea" or Djibouti* or Dominica* or Ecuador* or Egypt* or "El Salvador*" or Eritrea* or Eswatini* or Ethiopia* or Fiji or Gabon* or Gambia* or "Gaza Strip" or (Georgia NOT state) or Ghan* or Grenada or Guatemal* or (Guinea NOT ("guinea pig*")) or Guyan* or Haiti* or Herzegovin* or Hondur* or India* or Indones* or Iran* or Iraq* or "Ivory Coast" or Jamaica* or Jordan* or Kazakhstan* or Kenya* or Kiribat* or Kosov* or Kyrgyzstan* or Lao* or Leban* or Lesoth* or Liberia* or Libya* or Madagascar* or Malaw* or Malaysi* or Maldives or Mali or Malian or "Marshall Islands" or Mauritani* or Mauriti* or Mexic* or Micronesi* or Mocambiqu* or Moldov* or Mongoli* or Monteneg* or Montserrat or Morocc* or Mozambiqu* or Myanmar or Namibi* or Nauru or Nepal* or Nicaragu* or Niger or Nigeria* or Niue or "North Korea*" or "North Macedoni*" or Pakistan* or Palau* or Panam* or Paragua* or Peru* or Philippin* or Rhodesia* or Rwand* or "Saint Helena" or "Saint Lucia" or "Saint Vincent" or "St Helena" or "St Lucia" or "St Vincent" or Samoa* or "Sao Tome" or Senegal* or Serbia* or "Sierra Leon*" or "Solomon Islands" or Somali* or "South Africa*" or "Southern Africa*" or "Sri Lank*" or "Sub Saharan Africa*" or "Subsaharan Africa*" or Sudan* or Surinam* or Syria* or Tajikistan or Tanzani* or Thai* or "Timor-Leste" or Togo* or Tokelau or Tonga or Tunis* or Turki* or Turkmenistan or Tuvalu or Ugand* or Ukrain* or Uzbekistan or Vanuatu or Venezuel* or "Viet Nam*" or Vietnam* or "Wallis and Futuna" or "West Bank" or Yemen* or Zambia* or Zimbabw*).ab,ti,kf.** | 1,736,161 |
| #3 | **1 and 2** | 7,002 |
| #2 | **exp "Psychotic Disorders"/ or "Affective Disorders, Psychotic"/ or exp "Schizophrenia"/ or (psychot* or psychosis or psychoses or schizo*).ab,ti,kf.** | 323,087 |
| #1 | **"Early Medical Intervention"/ or ((early adj3 (intervention* or therap* or treatment* or detect* or recogni*)) or "coordinated specialty care" or "task-sharing" or "scalable intervention*").ab,ti,kf.** | 291,392 |

**Embase.com Search Results – 06 Feb 2024**

| **Search** | **Query** | **Results** |
| --- | --- | --- |
| #6 | **#5 NOT ('conference abstract'/it OR 'conference review'/it)** | 443 |
| #5 | **#3 AND #4** | 697 |
| #4 | **'developing country'/exp OR 'low income country'/exp OR 'middle income country'/exp OR ("developing countr*" OR "non-western" OR "nonwestern" OR "emerging econom*" OR "developing nation*" OR "developing population*" OR "developing econom*" OR "undeveloped countr*" OR "undeveloped nation*" OR "undeveloped economy" OR "undeveloped economies" OR "least developed countr*" OR "least developed nation*" OR "least developed economy" OR "least developed economies" OR "less-developed countr*" OR "less-developed nation*" OR "less-developed population" OR "less-developed populations" OR "less-developed econom*" OR "lesser developed countr*" OR "lesser developed nation*" OR "lesser developed population" OR "lesser developed populations" OR "lesser developed economy" OR "lesser developed economies" OR "under-developed countr*" OR "under-developed nation*" OR "underdeveloped countr*" OR "underdeveloped nation*" OR "underdeveloped population*" OR "underdeveloped econom*" OR "low income countr*" OR "middle income countr*" OR "low income nation*" OR "middle income nation*" OR "low income population*" OR "middle income population*" OR "low income econom*" OR "middle income econom*" OR "lower income countr*" OR "lower income nation*" OR "lower income population*" OR "lower income economy" OR "lower income economies" OR "resource limited" OR "low resource countr*" OR "lower resource countr*" OR "low resource nation*" OR "low resource population*" OR "low resource economy" OR "low resource economies" OR "underserved countr*" OR "underserved nation*" OR "underserved population*" OR "underserved economy" OR "underserved economies" OR "under-served country" OR "under-served countries" OR "under-served nation" OR "under-served nations" OR "under-served population" OR "under-served populations" OR "underserved economy" OR "underserved economies" OR "derived countr*" OR "deprived nation" OR "deprived nations" OR "derived population*" OR "deprived economy" OR "deprived economies" OR "poor countr*" OR "poor nation*" OR "poor population*" OR "poor econom*" OR "poorer countr*" OR "poorer nation*" OR "poorer population*" OR "poorer econom*" OR "lmic" OR "lmics" OR "lami" OR "transitional countr*" OR "transitional nation" OR "transitional nations" OR "transitional econom*" OR "transition countr*" OR "transition nation*" OR "transition econom*" OR "low resource setting*" OR "lower resource setting*" OR "middle resource setting*" OR "Third World*" OR Afghan* OR Alban* OR Algeria* OR Angol* OR Argentin* OR Armenia* OR Azerbaijan* OR Bangladesh* OR Belarus* OR Belize* OR Benin OR Bhutan* OR Bolivia* OR Bosnia* OR Botswan* OR Brazil* OR "Burkina Faso" OR Burundi* OR "Cabo Verd*" OR Cambodia* OR Cameroon* OR "Central African Republic" OR Chad OR China OR Colombia* OR Comoros OR Congo* OR "Costa Rica*" OR "Côte d Ivoire" OR Cuba* OR "Republic of Korea" OR Djibouti* OR Dominica* OR Ecuador* OR Egypt* OR "El Salvador*" OR Eritrea* OR Eswatini* OR Ethiopia* OR Fiji OR Gabon* OR Gambia* OR "Gaza Strip" OR (Georgia NOT state) OR Ghan* OR Grenada OR Guatemal* OR (Guinea NOT ("guinea pig*")) OR Guyan* OR Haiti* OR Herzegovin* OR Hondur* OR India* OR Indones* OR Iran* OR Iraq* OR "Ivory Coast" OR Jamaica* OR Jordan* OR Kazakhstan* OR Kenya* OR Kiribat* OR Kosov* OR Kyrgyzstan* OR Lao* OR Leban* OR Lesoth* OR Liberia* OR Libya* OR Madagascar* OR Malaw* OR Malaysi* OR Maldives OR Mali OR Malian OR "Marshall Islands" OR Mauritani* OR Mauriti* OR Mexic* OR Micronesi* OR Mocambiqu* OR Moldov* OR Mongoli* OR Monteneg* OR Montserrat OR Morocc* OR Mozambiqu* OR Myanmar OR Namibi* OR Nauru OR Nepal* OR Nicaragu* OR Niger OR Nigeria* OR Niue OR "North Korea*" OR "North Macedoni*" OR Pakistan* OR Palau* OR Panam* OR Paragua* OR Peru* OR Philippin* OR Rhodesia* OR Rwand* OR "Saint Helena" OR "Saint Lucia" OR "Saint Vincent" OR "St Helena" OR "St Lucia" OR "St Vincent" OR Samoa* OR "Sao Tome" OR Senegal* OR Serbia* OR "Sierra Leon*" OR "Solomon Islands" OR Somali* OR "South Africa*" OR "Southern Africa*" OR "Sri Lank*" OR "Sub Saharan Africa*" OR "Subsaharan Africa*" OR Sudan* OR Surinam* OR Syria* OR Tajikistan OR Tanzani* OR Thai* OR "Timor-Leste" OR Togo* OR Tokelau OR Tonga OR Tunis* OR Turki* OR Turkmenistan OR Tuvalu OR Ugand* OR Ukrain* OR Uzbekistan OR Vanuatu OR Venezuel* OR "Viet Nam*" OR Vietnam* OR "Wallis and Futuna" OR "West Bank" OR Yemen* OR Zambia* OR Zimbabw*):ab,ti,kw** | 2,200,058 |
| #3 | **#1 AND #2** | 13,963 |
| #2 | **'psychosis'/exp OR 'affective psychosis'/exp OR (psychot* OR psychosis OR psychoses OR schizo*):ab,ti,kw** | 519,246 |
| #1 | **'early intervention'/exp OR ((early NEAR/3 (intervention* OR therap* OR treatment* OR detect* OR recogni*)) OR "coordinated specialty care" OR "task-sharing" OR "scalable intervention*"):ab,ti,kw** | 443,147 |

**APA PsycInfo (Ebsco) Search Results – 06 Feb 2024**

| **Search** | **Query** | **Results** |
| --- | --- | --- |
| S5 | **S3 AND S4** | 300 |
| S4 | **DE "Developing Countries" OR DE "Emerging Economies" OR TI ("developing countr*" OR "non-western" OR "nonwestern" OR "emerging econom*" OR "developing nation*" OR "developing population*" OR "developing econom*" OR "undeveloped countr*" OR "undeveloped nation*" OR "undeveloped economy" OR "undeveloped economies" OR "least developed countr*" OR "least developed nation*" OR "least developed economy" OR "least developed economies" OR "less-developed countr*" OR "less-developed nation*" OR "less-developed population" OR "less-developed populations" OR "less-developed econom*" OR "lesser developed countr*" OR "lesser developed nation*" OR "lesser developed population" OR "lesser developed populations" OR "lesser developed economy" OR "lesser developed economies" OR "under-developed countr*" OR "under-developed nation*" OR "underdeveloped countr*" OR "underdeveloped nation*" OR "underdeveloped population*" OR "underdeveloped econom*" OR "low income countr*" OR "middle income countr*" OR "low income nation*" OR "middle income nation*" OR "low income population*" OR "middle income population*" OR "low income econom*" OR "middle income econom*" OR "lower income countr*" OR "lower income nation*" OR "lower income population*" OR "lower income economy" OR "lower income economies" OR "resource limited" OR "low resource countr*" OR "lower resource countr*" OR "low resource nation*" OR "low resource population*" OR "low resource economy" OR "low resource economies" OR "underserved countr*" OR "underserved nation*" OR "underserved population*" OR "underserved economy" OR "underserved economies" OR "under-served country" OR "under-served countries" OR "under-served nation" OR "under-served nations" OR "under-served population" OR "under-served populations" OR "underserved economy" OR "underserved economies" OR "derived countr*" OR "deprived nation" OR "deprived nations" OR "derived population*" OR "deprived economy" OR "deprived economies" OR "poor countr*" OR "poor nation*" OR "poor population*" OR "poor econom*" OR "poorer countr*" OR "poorer nation*" OR "poorer population*" OR "poorer econom*" OR "lmic" OR "lmics" OR "lami" OR "transitional countr*" OR "transitional nation" OR "transitional nations" OR "transitional econom*" OR "transition countr*" OR "transition nation*" OR "transition econom*" OR "low resource setting*" OR "lower resource setting*" OR "middle resource setting*" OR "Third World*" OR "Afghan*" OR "Alban*" OR "Algeria*" OR "Angol*" OR "Argentin*" OR "Armenia*" OR "Azerbaijan*" OR "Bangladesh*" OR "Belarus*" OR "Belize*" OR "Benin" OR "Bhutan*" OR "Bolivia*" OR "Bosnia*" OR "Botswan*" OR "Brazil*" OR "Burkina Faso" OR "Burundi*" OR "Cabo Verd*" OR "Cambodia*" OR "Cameroon*" OR "Central African Republic" OR "Chad" OR "China" OR "Colombia*" OR "Comoros" OR "Congo*" OR "Costa Rica*" OR "Côte d'Ivoire" OR "Cuba*" OR "Republic of Korea" OR "Djibouti*" OR "Dominica*" OR "Ecuador*" OR "Egypt*" OR "El Salvador*" OR "Eritrea*" OR "Eswatini*" OR "Ethiopia*" OR "Fiji" OR "Gabon*" OR "Gambia*" OR "Gaza Strip" OR ("Georgia" NOT "state") OR "Ghan*" OR "Grenada" OR "Guatemal*" OR ("Guinea" NOT ("guinea pig*")) OR "Guyan*" OR "Haiti*" OR "Herzegovin*" OR "Hondur*" OR "India*" OR "Indones*" OR "Iran*" OR "Iraq*" OR "Ivory Coast" OR "Jamaica*" OR "Jordan*" OR "Kazakhstan*" OR "Kenya*" OR "Kiribat*" OR "Kosov*" OR "Kyrgyzstan*" OR "Lao*" OR "Leban*" OR "Lesoth*" OR "Liberia*" OR "Libya*" OR "Madagascar*" OR "Malaw*" OR "Malaysi*" OR "Maldives" OR "Mali" OR "Malian" OR "Marshall Islands" OR "Mauritani*" OR "Mauriti*" OR "Mexic*" OR "Micronesi*" OR "Mocambiqu*" OR "Moldov*" OR "Mongoli*" OR "Monteneg*" OR "Montserrat" OR "Morocc*" OR "Mozambiqu*" OR "Myanmar" OR "Namibi*" OR "Nauru" OR "Nepal*" OR "Nicaragu*" OR "Niger" OR "Nigeria*" OR "Niue" OR "North Korea*" OR "North Macedoni*" OR "Pakistan*" OR "Palau*" OR "Panam*" OR "Paragua*" OR "Peru*" OR "Philippin*" OR "Rhodesia*" OR "Rwand*" OR "Saint Helena" OR "Saint Lucia" OR "Saint Vincent" OR "St Helena" OR "St Lucia" OR "St Vincent" OR "Samoa*" OR "Sao Tome" OR "Senegal*" OR "Serbia*" OR "Sierra Leon*" OR "Solomon Islands" OR "Somali*" OR "South Africa*" OR "Southern Africa*" OR "Sri Lank*" OR "Sub Saharan Africa*" OR "Subsaharan Africa*" OR "Sudan*" OR "Surinam*" OR "Syria*" OR "Tajikistan" OR "Tanzani*" OR "Thai*" OR "Timor-Leste" OR "Togo*" OR "Tokelau" OR "Tonga" OR "Tunis*" OR "Turki*" OR "Turkmenistan" OR "Tuvalu" OR "Ugand*" OR "Ukrain*" OR "Uzbekistan" OR "Vanuatu" OR "Venezuel*" OR "Viet Nam*" OR "Vietnam*" OR "Wallis and Futuna" OR "West Bank" OR "Yemen*" OR "Zambia*" OR "Zimbabw*") OR AB ("developing countr*" OR "non-western" OR "nonwestern" OR "emerging econom*" OR "developing nation*" OR "developing population*" OR "developing econom*" OR "undeveloped countr*" OR "undeveloped nation*" OR "undeveloped economy" OR "undeveloped economies" OR "least developed countr*" OR "least developed nation*" OR "least developed economy" OR "least developed economies" OR "less-developed countr*" OR "less-developed nation*" OR "less-developed population" OR "less-developed populations" OR "less-developed econom*" OR "lesser developed countr*" OR "lesser developed nation*" OR "lesser developed population" OR "lesser developed populations" OR "lesser developed economy" OR "lesser developed economies" OR "under-developed countr*" OR "under-developed nation*" OR "underdeveloped countr*" OR "underdeveloped nation*" OR "underdeveloped population*" OR "underdeveloped econom*" OR "low income countr*" OR "middle income countr*" OR "low income nation*" OR "middle income nation*" OR "low income population*" OR "middle income population*" OR "low income econom*" OR "middle income econom*" OR "lower income countr*" OR "lower income nation*" OR "lower income population*" OR "lower income economy" OR "lower income economies" OR "resource limited" OR "low resource countr*" OR "lower resource countr*" OR "low resource nation*" OR "low resource population*" OR "low resource economy" OR "low resource economies" OR "underserved countr*" OR "underserved nation*" OR "underserved population*" OR "underserved economy" OR "underserved economies" OR "under-served country" OR "under-served countries" OR "under-served nation" OR "under-served nations" OR "under-served population" OR "under-served populations" OR "underserved economy" OR "underserved economies" OR "derived countr*" OR "deprived nation" OR "deprived nations" OR "derived population*" OR "deprived economy" OR "deprived economies" OR "poor countr*" OR "poor nation*" OR "poor population*" OR "poor econom*" OR "poorer countr*" OR "poorer nation*" OR "poorer population*" OR "poorer econom*" OR "lmic" OR "lmics" OR "lami" OR "transitional countr*" OR "transitional nation" OR "transitional nations" OR "transitional econom*" OR "transition countr*" OR "transition nation*" OR "transition econom*" OR "low resource setting*" OR "lower resource setting*" OR "middle resource setting*" OR "Third World*" OR "Afghan*" OR "Alban*" OR "Algeria*" OR "Angol*" OR "Argentin*" OR "Armenia*" OR "Azerbaijan*" OR "Bangladesh*" OR "Belarus*" OR "Belize*" OR "Benin" OR "Bhutan*" OR "Bolivia*" OR "Bosnia*" OR "Botswan*" OR "Brazil*" OR "Burkina Faso" OR "Burundi*" OR "Cabo Verd*" OR "Cambodia*" OR "Cameroon*" OR "Central African Republic" OR "Chad" OR "China" OR "Colombia*" OR "Comoros" OR "Congo*" OR "Costa Rica*" OR "Côte d'Ivoire" OR "Cuba*" OR "Republic of Korea" OR "Djibouti*" OR "Dominica*" OR "Ecuador*" OR "Egypt*" OR "El Salvador*" OR "Eritrea*" OR "Eswatini*" OR "Ethiopia*" OR "Fiji" OR "Gabon*" OR "Gambia*" OR "Gaza Strip" OR ("Georgia" NOT "state") OR "Ghan*" OR "Grenada" OR "Guatemal*" OR ("Guinea" NOT ("guinea pig*")) OR "Guyan*" OR "Haiti*" OR "Herzegovin*" OR "Hondur*" OR "India*" OR "Indones*" OR "Iran*" OR "Iraq*" OR "Ivory Coast" OR "Jamaica*" OR "Jordan*" OR "Kazakhstan*" OR "Kenya*" OR "Kiribat*" OR "Kosov*" OR "Kyrgyzstan*" OR "Lao*" OR "Leban*" OR "Lesoth*" OR "Liberia*" OR "Libya*" OR "Madagascar*" OR "Malaw*" OR "Malaysi*" OR "Maldives" OR "Mali" OR "Malian" OR "Marshall Islands" OR "Mauritani*" OR "Mauriti*" OR "Mexic*" OR "Micronesi*" OR "Mocambiqu*" OR "Moldov*" OR "Mongoli*" OR "Monteneg*" OR "Montserrat" OR "Morocc*" OR "Mozambiqu*" OR "Myanmar" OR "Namibi*" OR "Nauru" OR "Nepal*" OR "Nicaragu*" OR "Niger" OR "Nigeria*" OR "Niue" OR "North Korea*" OR "North Macedoni*" OR "Pakistan*" OR "Palau*" OR "Panam*" OR "Paragua*" OR "Peru*" OR "Philippin*" OR "Rhodesia*" OR "Rwand*" OR "Saint Helena" OR "Saint Lucia" OR "Saint Vincent" OR "St Helena" OR "St Lucia" OR "St Vincent" OR "Samoa*" OR "Sao Tome" OR "Senegal*" OR "Serbia*" OR "Sierra Leon*" OR "Solomon Islands" OR "Somali*" OR "South Africa*" OR "Southern Africa*" OR "Sri Lank*" OR "Sub Saharan Africa*" OR "Subsaharan Africa*" OR "Sudan*" OR "Surinam*" OR "Syria*" OR "Tajikistan" OR "Tanzani*" OR "Thai*" OR "Timor-Leste" OR "Togo*" OR "Tokelau" OR "Tonga" OR "Tunis*" OR "Turki*" OR "Turkmenistan" OR "Tuvalu" OR "Ugand*" OR "Ukrain*" OR "Uzbekistan" OR "Vanuatu" OR "Venezuel*" OR "Viet Nam*" OR "Vietnam*" OR "Wallis and Futuna" OR "West Bank" OR "Yemen*" OR "Zambia*" OR "Zimbabw*") OR KW ("developing countr*" OR "non-western" OR "nonwestern" OR "emerging econom*" OR "developing nation*" OR "developing population*" OR "developing econom*" OR "undeveloped countr*" OR "undeveloped nation*" OR "undeveloped economy" OR "undeveloped economies" OR "least developed countr*" OR "least developed nation*" OR "least developed economy" OR "least developed economies" OR "less-developed countr*" OR "less-developed nation*" OR "less-developed population" OR "less-developed populations" OR "less-developed econom*" OR "lesser developed countr*" OR "lesser developed nation*" OR "lesser developed population" OR "lesser developed populations" OR "lesser developed economy" OR "lesser developed economies" OR "under-developed countr*" OR "under-developed nation*" OR "underdeveloped countr*" OR "underdeveloped nation*" OR "underdeveloped population*" OR "underdeveloped econom*" OR "low income countr*" OR "middle income countr*" OR "low income nation*" OR "middle income nation*" OR "low income population*" OR "middle income population*" OR "low income econom*" OR "middle income econom*" OR "lower income countr*" OR "lower income nation*" OR "lower income population*" OR "lower income economy" OR "lower income economies" OR "resource limited" OR "low resource countr*" OR "lower resource countr*" OR "low resource nation*" OR "low resource population*" OR "low resource economy" OR "low resource economies" OR "underserved countr*" OR "underserved nation*" OR "underserved population*" OR "underserved economy" OR "underserved economies" OR "under-served country" OR "under-served countries" OR "under-served nation" OR "under-served nations" OR "under-served population" OR "under-served populations" OR "underserved economy" OR "underserved economies" OR "derived countr*" OR "deprived nation" OR "deprived nations" OR "derived population*" OR "deprived economy" OR "deprived economies" OR "poor countr*" OR "poor nation*" OR "poor population*" OR "poor econom*" OR "poorer countr*" OR "poorer nation*" OR "poorer population*" OR "poorer econom*" OR "lmic" OR "lmics" OR "lami" OR "transitional countr*" OR "transitional nation" OR "transitional nations" OR "transitional econom*" OR "transition countr*" OR "transition nation*" OR "transition econom*" OR "low resource setting*" OR "lower resource setting*" OR "middle resource setting*" OR "Third World*" OR "Afghan*" OR "Alban*" OR "Algeria*" OR "Angol*" OR "Argentin*" OR "Armenia*" OR "Azerbaijan*" OR "Bangladesh*" OR "Belarus*" OR "Belize*" OR "Benin" OR "Bhutan*" OR "Bolivia*" OR "Bosnia*" OR "Botswan*" OR "Brazil*" OR "Burkina Faso" OR "Burundi*" OR "Cabo Verd*" OR "Cambodia*" OR "Cameroon*" OR "Central African Republic" OR "Chad" OR "China" OR "Colombia*" OR "Comoros" OR "Congo*" OR "Costa Rica*" OR "Côte d'Ivoire" OR "Cuba*" OR "Republic of Korea" OR "Djibouti*" OR "Dominica*" OR "Ecuador*" OR "Egypt*" OR "El Salvador*" OR "Eritrea*" OR "Eswatini*" OR "Ethiopia*" OR "Fiji" OR "Gabon*" OR "Gambia*" OR "Gaza Strip" OR ("Georgia" NOT "state") OR "Ghan*" OR "Grenada" OR "Guatemal*" OR ("Guinea" NOT ("guinea pig*")) OR "Guyan*" OR "Haiti*" OR "Herzegovin*" OR "Hondur*" OR "India*" OR "Indones*" OR "Iran*" OR "Iraq*" OR "Ivory Coast" OR "Jamaica*" OR "Jordan*" OR "Kazakhstan*" OR "Kenya*" OR "Kiribat*" OR "Kosov*" OR "Kyrgyzstan*" OR "Lao*" OR "Leban*" OR "Lesoth*" OR "Liberia*" OR "Libya*" OR "Madagascar*" OR "Malaw*" OR "Malaysi*" OR "Maldives" OR "Mali" OR "Malian" OR "Marshall Islands" OR "Mauritani*" OR "Mauriti*" OR "Mexic*" OR "Micronesi*" OR "Mocambiqu*" OR "Moldov*" OR "Mongoli*" OR "Monteneg*" OR "Montserrat" OR "Morocc*" OR "Mozambiqu*" OR "Myanmar" OR "Namibi*" OR "Nauru" OR "Nepal*" OR "Nicaragu*" OR "Niger" OR "Nigeria*" OR "Niue" OR "North Korea*" OR "North Macedoni*" OR "Pakistan*" OR "Palau*" OR "Panam*" OR "Paragua*" OR "Peru*" OR "Philippin*" OR "Rhodesia*" OR "Rwand*" OR "Saint Helena" OR "Saint Lucia" OR "Saint Vincent" OR "St Helena" OR "St Lucia" OR "St Vincent" OR "Samoa*" OR "Sao Tome" OR "Senegal*" OR "Serbia*" OR "Sierra Leon*" OR "Solomon Islands" OR "Somali*" OR "South Africa*" OR "Southern Africa*" OR "Sri Lank*" OR "Sub Saharan Africa*" OR "Subsaharan Africa*" OR "Sudan*" OR "Surinam*" OR "Syria*" OR "Tajikistan" OR "Tanzani*" OR "Thai*" OR "Timor-Leste" OR "Togo*" OR "Tokelau" OR "Tonga" OR "Tunis*" OR "Turki*" OR "Turkmenistan" OR "Tuvalu" OR "Ugand*" OR "Ukrain*" OR "Uzbekistan" OR "Vanuatu" OR "Venezuel*" OR "Viet Nam*" OR "Vietnam*" OR "Wallis and Futuna" OR "West Bank" OR "Yemen*" OR "Zambia*" OR "Zimbabw*")** | 313,503 |
| S3 | **S1 AND S2** | 7,876 |
| S2 | **DE "Psychosis" OR DE "Affective Psychosis" OR DE "Chronic Psychosis" OR DE "Schizophrenia" OR TI ("psychot*" OR "psychosis" OR "psychoses" OR "schizo*") OR AB ("psychot*" OR "psychosis" OR "psychoses" OR "schizo*") OR KW ("psychot*" OR "psychosis" OR "psychoses" OR "schizo*")** | 339,949 |
| S1 | **DE "Early Intervention" OR TI (("early" N3 ("intervention*" OR "therap*" OR "treatment*" OR "detect*" OR "recogni*")) OR "coordinated specialty care" OR "task-sharing" OR "scalable intervention*") OR AB (("early" N3 ("intervention*" OR "therap*" OR "treatment*" OR "detect*" OR "recogni*")) OR "coordinated specialty care" OR "task-sharing" OR "scalable intervention*") OR KW (("early" N3 ("intervention*" OR "therap*" OR "treatment*" OR "detect*" OR "recogni*")) OR "coordinated specialty care" OR "task-sharing" OR "scalable intervention*")** | 54,158 |

**Web of Science (Core Collection) Search Results – 06 Feb 2024**

| **Search** | **Query** | **Results** |
| --- | --- | --- |
| #5 | **#3 AND #4** | 493 |
| #4 | **TS=("developing countr*" OR "non-western" OR "nonwestern" OR "emerging econom*" OR "developing nation*" OR "developing population*" OR "developing econom*" OR "undeveloped countr*" OR "undeveloped nation*" OR "undeveloped economy" OR "undeveloped economies" OR "least developed countr*" OR "least developed nation*" OR "least developed economy" OR "least developed economies" OR "less-developed countr*" OR "less-developed nation*" OR "less-developed population" OR "less-developed populations" OR "less-developed econom*" OR "lesser developed countr*" OR "lesser developed nation*" OR "lesser developed population" OR "lesser developed populations" OR "lesser developed economy" OR "lesser developed economies" OR "under-developed countr*" OR "under-developed nation*" OR "underdeveloped countr*" OR "underdeveloped nation*" OR "underdeveloped population*" OR "underdeveloped econom*" OR "low income countr*" OR "middle income countr*" OR "low income nation*" OR "middle income nation*" OR "low income population*" OR "middle income population*" OR "low income econom*" OR "middle income econom*" OR "lower income countr*" OR "lower income nation*" OR "lower income population*" OR "lower income economy" OR "lower income economies" OR "resource limited" OR "low resource countr*" OR "lower resource countr*" OR "low resource nation*" OR "low resource population*" OR "low resource economy" OR "low resource economies" OR "underserved countr*" OR "underserved nation*" OR "underserved population*" OR "underserved economy" OR "underserved economies" OR "under-served country" OR "under-served countries" OR "under-served nation" OR "under-served nations" OR "under-served population" OR "under-served populations" OR "underserved economy" OR "underserved economies" OR "derived countr*" OR "deprived nation" OR "deprived nations" OR "derived population*" OR "deprived economy" OR "deprived economies" OR "poor countr*" OR "poor nation*" OR "poor population*" OR "poor econom*" OR "poorer countr*" OR "poorer nation*" OR "poorer population*" OR "poorer econom*" OR "lmic" OR "lmics" OR "lami" OR "transitional countr*" OR "transitional nation" OR "transitional nations" OR "transitional econom*" OR "transition countr*" OR "transition nation*" OR "transition econom*" OR "low resource setting*" OR "lower resource setting*" OR "middle resource setting*" OR "Third World*" OR "Afghan*" OR "Alban*" OR "Algeria*" OR "Angol*" OR "Argentin*" OR "Armenia*" OR "Azerbaijan*" OR "Bangladesh*" OR "Belarus*" OR "Belize*" OR "Benin" OR "Bhutan*" OR "Bolivia*" OR "Bosnia*" OR "Botswan*" OR "Brazil*" OR "Burkina Faso" OR "Burundi*" OR "Cabo Verd*" OR "Cambodia*" OR "Cameroon*" OR "Central African Republic" OR "Chad" OR "China" OR "Colombia*" OR "Comoros" OR "Congo*" OR "Costa Rica*" OR "Côte d'Ivoire" OR "Cuba*" OR "Republic of Korea" OR "Djibouti*" OR "Dominica*" OR "Ecuador*" OR "Egypt*" OR "El Salvador*" OR "Eritrea*" OR "Eswatini*" OR "Ethiopia*" OR "Fiji" OR "Gabon*" OR "Gambia*" OR "Gaza Strip" OR ("Georgia" NOT "state") OR "Ghan*" OR "Grenada" OR "Guatemal*" OR ("Guinea" NOT ("guinea pig*")) OR "Guyan*" OR "Haiti*" OR "Herzegovin*" OR "Hondur*" OR "India*" OR "Indones*" OR "Iran*" OR "Iraq*" OR "Ivory Coast" OR "Jamaica*" OR "Jordan*" OR "Kazakhstan*" OR "Kenya*" OR "Kiribat*" OR "Kosov*" OR "Kyrgyzstan*" OR "Lao*" OR "Leban*" OR "Lesoth*" OR "Liberia*" OR "Libya*" OR "Madagascar*" OR "Malaw*" OR "Malaysi*" OR "Maldives" OR "Mali" OR "Malian" OR "Marshall Islands" OR "Mauritani*" OR "Mauriti*" OR "Mexic*" OR "Micronesi*" OR "Mocambiqu*" OR "Moldov*" OR "Mongoli*" OR "Monteneg*" OR "Montserrat" OR "Morocc*" OR "Mozambiqu*" OR "Myanmar" OR "Namibi*" OR "Nauru" OR "Nepal*" OR "Nicaragu*" OR "Niger" OR "Nigeria*" OR "Niue" OR "North Korea*" OR "North Macedoni*" OR "Pakistan*" OR "Palau*" OR "Panam*" OR "Paragua*" OR "Peru*" OR "Philippin*" OR "Rhodesia*" OR "Rwand*" OR "Saint Helena" OR "Saint Lucia" OR "Saint Vincent" OR "St Helena" OR "St Lucia" OR "St Vincent" OR "Samoa*" OR "Sao Tome" OR "Senegal*" OR "Serbia*" OR "Sierra Leon*" OR "Solomon Islands" OR "Somali*" OR "South Africa*" OR "Southern Africa*" OR "Sri Lank*" OR "Sub Saharan Africa*" OR "Subsaharan Africa*" OR "Sudan*" OR "Surinam*" OR "Syria*" OR "Tajikistan" OR "Tanzani*" OR "Thai*" OR "Timor-Leste" OR "Togo*" OR "Tokelau" OR "Tonga" OR "Tunis*" OR "Turki*" OR "Turkmenistan" OR "Tuvalu" OR "Ugand*" OR "Ukrain*" OR "Uzbekistan" OR "Vanuatu" OR "Venezuel*" OR "Viet Nam*" OR "Vietnam*" OR "Wallis and Futuna" OR "West Bank" OR "Yemen*" OR "Zambia*" OR "Zimbabw*")** | 4,538,485 |
| #3 | **#1 AND #2** | 10,026 |
| #2 | **TS=("psychot*" OR "psychosis" OR "psychoses" OR "schizo*")** | 432,174 |
| #1 | **TS=(("early" NEAR/3 ("intervention*" OR "therap*" OR "treatment*" OR "detect*" OR "recogni*")) OR "coordinated specialty care" OR "task-sharing" OR "scalable intervention*")** | 329,787 |

**Scopus Search Results – 06 Feb 2024**

| **Search** | **Query** | **Results** |
| --- | --- | --- |
| #5 | **#3 AND #4** | 456 |
| #4 | **TITLE-ABS ("developing countr*" OR "non-western" OR "nonwestern" OR "emerging econom*" OR "developing nation*" OR "developing population*" OR "developing econom*" OR "undeveloped countr*" OR "undeveloped nation*" OR "undeveloped economy" OR "undeveloped economies" OR "least developed countr*" OR "least developed nation*" OR "least developed economy" OR "least developed economies" OR "less-developed countr*" OR "less-developed nation*" OR "less-developed population" OR "less-developed populations" OR "less-developed econom*" OR "lesser developed countr*" OR "lesser developed nation*" OR "lesser developed population" OR "lesser developed populations" OR "lesser developed economy" OR "lesser developed economies" OR "under-developed countr*" OR "under-developed nation*" OR "underdeveloped countr*" OR "underdeveloped nation*" OR "underdeveloped population*" OR "underdeveloped econom*" OR "low income countr*" OR "middle income countr*" OR "low income nation*" OR "middle income nation*" OR "low income population*" OR "middle income population*" OR "low income econom*" OR "middle income econom*" OR "lower income countr*" OR "lower income nation*" OR "lower income population*" OR "lower income economy" OR "lower income economies" OR "resource limited" OR "low resource countr*" OR "lower resource countr*" OR "low resource nation*" OR "low resource population*" OR "low resource economy" OR "low resource economies" OR "underserved countr*" OR "underserved nation*" OR "underserved population*" OR "underserved economy" OR "underserved economies" OR "under-served country" OR "under-served countries" OR "under-served nation" OR "under-served nations" OR "under-served population" OR "under-served populations" OR "underserved economy" OR "underserved economies" OR "derived countr*" OR "deprived nation" OR "deprived nations" OR "derived population*" OR "deprived economy" OR "deprived economies" OR "poor countr*" OR "poor nation*" OR "poor population*" OR "poor econom*" OR "poorer countr*" OR "poorer nation*" OR "poorer population*" OR "poorer econom*" OR "lmic" OR "lmics" OR "lami" OR "transitional countr*" OR "transitional nation" OR "transitional nations" OR "transitional econom*" OR "transition countr*" OR "transition nation*" OR "transition econom*" OR "low resource setting*" OR "lower resource setting*" OR "middle resource setting*" OR "Third World*" OR "Afghan*" OR "Alban*" OR "Algeria*" OR "Angol*" OR "Argentin*" OR "Armenia*" OR "Azerbaijan*" OR "Bangladesh*" OR "Belarus*" OR "Belize*" OR "Benin" OR "Bhutan*" OR "Bolivia*" OR "Bosnia*" OR "Botswan*" OR "Brazil*" OR "Burkina Faso" OR "Burundi*" OR "Cabo Verd*" OR "Cambodia*" OR "Cameroon*" OR "Central African Republic" OR "Chad" OR "China" OR "Colombia*" OR "Comoros" OR "Congo*" OR "Costa Rica*" OR "Côte d'Ivoire" OR "Cuba*" OR "Republic of Korea" OR "Djibouti*" OR "Dominica*" OR "Ecuador*" OR "Egypt*" OR "El Salvador*" OR "Eritrea*" OR "Eswatini*" OR "Ethiopia*" OR "Fiji" OR "Gabon*" OR "Gambia*" OR "Gaza Strip" OR ("Georgia" ANDNOT "state") OR "Ghan*" OR "Grenada" OR "Guatemal*" OR ("Guinea" ANDNOT ("guinea pig*")) OR "Guyan*" OR "Haiti*" OR "Herzegovin*" OR "Hondur*" OR "India*" OR "Indones*" OR "Iran*" OR "Iraq*" OR "Ivory Coast" OR "Jamaica*" OR "Jordan*" OR "Kazakhstan*" OR "Kenya*" OR "Kiribat*" OR "Kosov*" OR "Kyrgyzstan*" OR "Lao*" OR "Leban*" OR "Lesoth*" OR "Liberia*" OR "Libya*" OR "Madagascar*" OR "Malaw*" OR "Malaysi*" OR "Maldives" OR "Mali" OR "Malian" OR "Marshall Islands" OR "Mauritani*" OR "Mauriti*" OR "Mexic*" OR "Micronesi*" OR "Mocambiqu*" OR "Moldov*" OR "Mongoli*" OR "Monteneg*" OR "Montserrat" OR "Morocc*" OR "Mozambiqu*" OR "Myanmar" OR "Namibi*" OR "Nauru" OR "Nepal*" OR "Nicaragu*" OR "Niger" OR "Nigeria*" OR "Niue" OR "North Korea*" OR "North Macedoni*" OR "Pakistan*" OR "Palau*" OR "Panam*" OR "Paragua*" OR "Peru*" OR "Philippin*" OR "Rhodesia*" OR "Rwand*" OR "Saint Helena" OR "Saint Lucia" OR "Saint Vincent" OR "St Helena" OR "St Lucia" OR "St Vincent" OR "Samoa*" OR "Sao Tome" OR "Senegal*" OR "Serbia*" OR "Sierra Leon*" OR "Solomon Islands" OR "Somali*" OR "South Africa*" OR "Southern Africa*" OR "Sri Lank*" OR "Sub Saharan Africa*" OR "Subsaharan Africa*" OR "Sudan*" OR "Surinam*" OR "Syria*" OR "Tajikistan" OR "Tanzani*" OR "Thai*" OR "Timor-Leste" OR "Togo*" OR "Tokelau" OR "Tonga" OR "Tunis*" OR "Turki*" OR "Turkmenistan" OR "Tuvalu" OR "Ugand*" OR "Ukrain*" OR "Uzbekistan" OR "Vanuatu" OR "Venezuel*" OR "Viet Nam*" OR "Vietnam*" OR "Wallis and Futuna" OR "West Bank" OR "Yemen*" OR "Zambia*" OR "Zimbabw*") OR AUTHKEY ("developing countr*" OR "non-western" OR "nonwestern" OR "emerging econom*" OR "developing nation*" OR "developing population*" OR "developing econom*" OR "undeveloped countr*" OR "undeveloped nation*" OR "undeveloped economy" OR "undeveloped economies" OR "least developed countr*" OR "least developed nation*" OR "least developed economy" OR "least developed economies" OR "less-developed countr*" OR "less-developed nation*" OR "less-developed population" OR "less-developed populations" OR "less-developed econom*" OR "lesser developed countr*" OR "lesser developed nation*" OR "lesser developed population" OR "lesser developed populations" OR "lesser developed economy" OR "lesser developed economies" OR "under-developed countr*" OR "under-developed nation*" OR "underdeveloped countr*" OR "underdeveloped nation*" OR "underdeveloped population*" OR "underdeveloped econom*" OR "low income countr*" OR "middle income countr*" OR "low income nation*" OR "middle income nation*" OR "low income population*" OR "middle income population*" OR "low income econom*" OR "middle income econom*" OR "lower income countr*" OR "lower income nation*" OR "lower income population*" OR "lower income economy" OR "lower income economies" OR "resource limited" OR "low resource countr*" OR "lower resource countr*" OR "low resource nation*" OR "low resource population*" OR "low resource economy" OR "low resource economies" OR "underserved countr*" OR "underserved nation*" OR "underserved population*" OR "underserved economy" OR "underserved economies" OR "under-served country" OR "under-served countries" OR "under-served nation" OR "under-served nations" OR "under-served population" OR "under-served populations" OR "underserved economy" OR "underserved economies" OR "derived countr*" OR "deprived nation" OR "deprived nations" OR "derived population*" OR "deprived economy" OR "deprived economies" OR "poor countr*" OR "poor nation*" OR "poor population*" OR "poor econom*" OR "poorer countr*" OR "poorer nation*" OR "poorer population*" OR "poorer econom*" OR "lmic" OR "lmics" OR "lami" OR "transitional countr*" OR "transitional nation" OR "transitional nations" OR "transitional econom*" OR "transition countr*" OR "transition nation*" OR "transition econom*" OR "low resource setting*" OR "lower resource setting*" OR "middle resource setting*" OR "Third World*" OR "Afghan*" OR "Alban*" OR "Algeria*" OR "Angol*" OR "Argentin*" OR "Armenia*" OR "Azerbaijan*" OR "Bangladesh*" OR "Belarus*" OR "Belize*" OR "Benin" OR "Bhutan*" OR "Bolivia*" OR "Bosnia*" OR "Botswan*" OR "Brazil*" OR "Burkina Faso" OR "Burundi*" OR "Cabo Verd*" OR "Cambodia*" OR "Cameroon*" OR "Central African Republic" OR "Chad" OR "China" OR "Colombia*" OR "Comoros" OR "Congo*" OR "Costa Rica*" OR "Côte d'Ivoire" OR "Cuba*" OR "Republic of Korea" OR "Djibouti*" OR "Dominica*" OR "Ecuador*" OR "Egypt*" OR "El Salvador*" OR "Eritrea*" OR "Eswatini*" OR "Ethiopia*" OR "Fiji" OR "Gabon*" OR "Gambia*" OR "Gaza Strip" OR ("Georgia" ANDNOT "state") OR "Ghan*" OR "Grenada" OR "Guatemal*" OR ("Guinea" ANDNOT ("guinea pig*")) OR "Guyan*" OR "Haiti*" OR "Herzegovin*" OR "Hondur*" OR "India*" OR "Indones*" OR "Iran*" OR "Iraq*" OR "Ivory Coast" OR "Jamaica*" OR "Jordan*" OR "Kazakhstan*" OR "Kenya*" OR "Kiribat*" OR "Kosov*" OR "Kyrgyzstan*" OR "Lao*" OR "Leban*" OR "Lesoth*" OR "Liberia*" OR "Libya*" OR "Madagascar*" OR "Malaw*" OR "Malaysi*" OR "Maldives" OR "Mali" OR "Malian" OR "Marshall Islands" OR "Mauritani*" OR "Mauriti*" OR "Mexic*" OR "Micronesi*" OR "Mocambiqu*" OR "Moldov*" OR "Mongoli*" OR "Monteneg*" OR "Montserrat" OR "Morocc*" OR "Mozambiqu*" OR "Myanmar" OR "Namibi*" OR "Nauru" OR "Nepal*" OR "Nicaragu*" OR "Niger" OR "Nigeria*" OR "Niue" OR "North Korea*" OR "North Macedoni*" OR "Pakistan*" OR "Palau*" OR "Panam*" OR "Paragua*" OR "Peru*" OR "Philippin*" OR "Rhodesia*" OR "Rwand*" OR "Saint Helena" OR "Saint Lucia" OR "Saint Vincent" OR "St Helena" OR "St Lucia" OR "St Vincent" OR "Samoa*" OR "Sao Tome" OR "Senegal*" OR "Serbia*" OR "Sierra Leon*" OR "Solomon Islands" OR "Somali*" OR "South Africa*" OR "Southern Africa*" OR "Sri Lank*" OR "Sub Saharan Africa*" OR "Subsaharan Africa*" OR "Sudan*" OR "Surinam*" OR "Syria*" OR "Tajikistan" OR "Tanzani*" OR "Thai*" OR "Timor-Leste" OR "Togo*" OR "Tokelau" OR "Tonga" OR "Tunis*" OR "Turki*" OR "Turkmenistan" OR "Tuvalu" OR "Ugand*" OR "Ukrain*" OR "Uzbekistan" OR "Vanuatu" OR "Venezuel*" OR "Viet Nam*" OR "Vietnam*" OR "Wallis and Futuna" OR "West Bank" OR "Yemen*" OR "Zambia*" OR "Zimbabw*")** | 5,730,374 |
| #3 | **#1 AND #2** | 9,992 |
| #2 | **TITLE-ABS ("psychot*" OR "psychosis" OR "psychoses" OR "schizo*") OR AUTHKEY ("psychot*" OR "psychosis" OR "psychoses" OR "schizo*")** | 408,797 |
| #1 | **TITLE-ABS (("early" W/3 ("intervention*" OR "therap*" OR "treatment*" OR "detect*" OR "recogni*")) OR "coordinated specialty care" OR "task-sharing" OR "scalable intervention*") OR AUTHKEY (("early" W/3 ("intervention*" OR "therap*" OR "treatment*" OR "detect*" OR "recogni*")) OR "coordinated specialty care" OR "task-sharing" OR "scalable intervention*")** | 490,078 |
